# Integrated survey of helminthic neglected tropical diseases and comparison of two mosquito sampling methods for lymphatic filariasis molecular xenomonitoring in the River Galana area, Kilifi County, coastal Kenya

**DOI:** 10.1371/journal.pone.0278655

**Published:** 2022-12-09

**Authors:** Sammy M. Njenga, Henry M. Kanyi, Cassian M. Mwatele, Dunstan A. Mukoko, Moses J. Bockarie, Louise A. Kelly-Hope

**Affiliations:** 1 Eastern and Southern Africa Centre of International Parasite Control, Kenya Medical Research Institute, Nairobi, Kenya; 2 Division of Vector Borne and Neglected Tropical Diseases, Ministry of Health, Nairobi, Kenya; 3 School of Community Health Sciences, Njala University, Bo, Sierra Leone; 4 Department of Tropical Disease Biology, Centre for Neglected Tropical Diseases, Liverpool School of Tropical Medicine, Liverpool, United Kingdom; Jazan University, SAUDI ARABIA

## Abstract

A lymphatic filariasis (LF) endemic focus along the River Galana/ Sabaki in Kilifi County, coastal Kenya, provided a platform to conduct an integrated survey for three helminthic neglected tropical diseases (NTDs), namely soil-transmitted helminthiasis (STH), schistosomiasis (SCH) and LF. Additionally, the study compared the performance of two mosquito trapping methods for LF molecular xenomonitoring (MX). Cross-sectional surveys measuring STH, SCH and LF prevalence were conducted in four villages. Mosquitoes were trapped using the CDC light trap (CDC-LT) and the Ifakara A tent trap (Ifakara-TT) methods and stored in pools which were tested for *Wuchereria bancrofti* DNA using the real-time polymerase chain reaction assay. A total of 907 people (436 adults; 471 children) participated in the parasitological testing. Among the STH infections, *Trichuris trichiura* and hookworms were most prevalent among the children and adult populations, respectively. The schistosome worm eggs detected belonged to the species *Schistosoma haematobium* and the prevalence of the infection was generally higher among the children compared with the adult population. The prevalence of LF infection among the adult population ranged from 1.8% to 7.6% across all 4 villages (P < 0.05). A total of 3,652 mosquitoes, including *Anopheles*, *Culex*, *Mansonia*, and *Aedes* species were collected. One mosquito pool consisting of *Anopheles* mosquitoes tested positive for filarial DNA out of 1,055 pools that were tested. The CDC-LT caught significantly more mosquitoes compared with the Ifakara-TT (P < 0.001). This study demonstrated that integrated epidemiological surveys using standard parasitological and entomological methods can provide useful information on co-endemic parasitic diseases which could help direct interventions and surveillance activities.

## Introduction

Neglected tropical diseases (NTDs) occur in poor communities and are associated with high levels of morbidity and stigma. Soil-transmitted helminthiasis (STH), schistosomiasis (SCH) and lymphatic filariasis (LF) are co-endemic NTDs in many countries in the sub-Saharan Africa region and targeted for control and/or elimination, primarily through preventive chemotherapy administered to populations, as part of the World Health Organization (WHO) NTD Roadmap 2021–2030 [1].

Soil-transmitted helminth infections are among the most prevalent infections with more than 1.5 billion people, or 24% of the world’s population, infected with STH infections worldwide [2]. The main species that infect humans are the roundworm (*Ascaris lumbricoides*), the whipworm (*Trichuris trichiura*) and hookworms (*Necator americanus* and *Ancylostoma duodenale*). Schistosomiasis affects over 200 million people worldwide with more than 90% living in the sub-Saharan Africa region where *Schistosoma mansoni* and *S*. *haematobium* are the most common species [3,4]. Lymphatic filariasis (LF) is a mosquito-borne parasitic infection caused by the filarial nematodes *Wuchereria bancrofti*, *Brugia malayi*, and *Brugia timori* [5]. However, *W*. *bancrofti* is the only known aetiologic agent of the disease in the sub-Saharan Africa region.

In Kenya, STH, SCH and LF are co-endemic in many areas of the coastal region. Urogenital SCH caused by *S*. *haematobium* is the principal type of schistosome infection in the region with intestinal SCH only known to occur in parts of Taita Taveta County. The area along the River Galana in Kilifi County, Kenya is historically known to be endemic for LF caused by *W*. *bancrofti* [6]. This site was among the first areas to receive mass drug administration (MDA) against LF in 2002, consisting of the co-administration of diethylcarbamazine citrate (DEC, 6 mg/kg) and albendazole (400 mg) as recommended by the WHO’s Global Programme to Eliminate Lymphatic Filariasis (GPELF) [7]. This area received four MDA rounds (non-consecutive) between 2002 and 2008, and in 2012 funding was provided to conduct an impact assessment. Since albendazole is a broad anthelminthic benzimidazole that also treats STH infections, it is likely that the LF MDA may have reduced *Ascaris lumbricoides*, *Trichuris trichiura* and hookworm infections [8,9]. However, the status of these STHs and other infections such as SCH were largely unknown in this area prior to the start of the MDA campaigns.

Integrated disease surveys are needed to inform multi-disease control programmes [10]. Integration is considered a cost-effective approach for maximizing human and financial resources in co-endemic areas with coordinated prevalence surveys, MDA and surveillance activities. The LF impact assessment in the River Galana area provided a platform to conduct an integrated survey to include STH, SCH and LF using a number of diagnostic tools. The WHO currently recommends the use of parasitological methods for the diagnosis of STH and SCH infections (Kato-Katz for STHs and intestinal SCH, and urine filtration for urogenital SCH) [11]. For LF testing, microscopy to detect microfilariae (MF) in nighttime blood and a rapid diagnostic test which detects circulating filarial antigen (CFA) in human blood are the standard diagnostic methods [12,13]. However, the use of entomological sampling and molecular detection of filarial DNA in mosquitoes, known as molecular xenomonitoring (MX) is also considered a surveillance tool for LF that may have particular utility in low endemic settings, and could supplement the standard diagnostics [14,15]. Results of a systematic review of alternative surveillance approaches for LF in low prevalence settings suggest that sensitivity of LF surveillance can be increased by incorporating newer human diagnostic tests (including antibody tests) and the use of mosquito MX may be able to help identify and target areas of active transmission [15].

Various commercial mosquito traps for sampling and surveillance of mosquito-borne pathogens are available. One of the most commonly employed tools for catching host-seeking malaria vectors is the Centers for Disease Control and Prevention (CDC) miniature light trap (CDC-LT), which is typically positioned indoors near an occupied bed area [16]. However, the widespread use of indoor-targeted insecticidal based interventions such as long-lasting insecticide-treated nets (LLINs) and indoor residual spraying (IRS) have drastically reduced endophilic and endophagic mosquito vectors [17]. Consequently, traps for capturing host-seeking mosquitoes outside of houses are suggested to be more suitable for sampling the exophagic vectors. The Ifakara A tent trap (Ifakara-TT) has been proposed as a relatively sensitive and practical method for sampling mosquito vectors outdoors [18,19].

The main aim of this study was to assess the feasibility of using LF impact assessment as a platform to conduct an integrated survey in adult and schoolchildren population groups and to determine the value of this strategy for multi-disease surveillance targeting STH, SCH and LF in four selected villages in the River Galana area where four MDA rounds of DEC and albendazole had been administered. A secondary aim was to evaluate the field performance of the CDC-LT and Ifakara-TT, and evaluate MX as a complementary tool for the monitoring and surveillance of LF.

## Materials and methods

### Study area and design

To determine the prevalence of STH, SCH and LF along the River Galana (also known as Sabaki) area in Malindi, a community- and school-based cross-sectional survey was conducted in adults and children, respectively in four rural villages, namely Jilore, Marikano, Magongoloni and Mkondoni in 2012. The study setting is known to be endemic for LF and has previously been described in detail [20,21]. The baseline prevalence of microfilaraemia and antigenaemia (using ICT test) in the area in 2002 ranged from 17.7% to 22.9% and 27.5% to 42.4%, respectively [20]. The population had previously received a total of four MDA rounds for LF between 2002 and 2008 covering at least 65% of the population at risk [22,23], but not given every year as recommended by the WHO.

The people inhabiting the study area are the Giriama, a sub-group of the Mijikenda ethnic group who usually live in homesteads widely dispersed in the countryside. The homesteads are in turn administratively organized in villages and one or two villages are served by a primary school situated in the village. The Giriama practice small-scale farming and their houses are mainly made of mud walls and thatched with coconut leaves (makuti), grass, or iron sheets with open eaves that can allow entry of mosquitoes. A previous study was conducted in this setting to evaluate the mosquito vector abundance and diversity in urban, peri-urban, and rural areas [24]. The study found that mosquitoes belonging to the genus *Anopheles* (mainly *An*. *gambiae*, *An*. *funestus* and *An*. *Coustani*) were predominant in the rural areas while *Aedes aegypti* and *Culex quinquefasciatus* were mostly found in urban and peri-urban areas. In addition, mosquito distribution varied significantly in each collection site.

### Sample size

The Cochran equation [25] for determining sample size of surveys based on LF which provided a platform for the surveys was used:

$$\text{n}=\left({\text{Z}}^{2}\text{pq}\right)/{\text{e}}^{2}$$

Where, n is the sample size, Z is the abscissa of the normal curve that cuts off at an area α (value of Z = 1.96 for 95% confidence level), p is the assumed (expected) prevalence, and q is 1 –p. Finally, e is the desired level of precision (0.03 was used in this study).

It was assumed that the expected prevalence of LF infection in the area was about 5% following the mass treatments. The computed n was 203 individuals. However, a 15% loss to follow up was assumed which resulted in n being 233 individuals for each arm. Since the individuals were spread over 4 villages, a design effect of 2 was applied to account for the community-level clustering of positive cases. Therefore, n = 233 x 2 = 466 study participants in each study arm.

There was no specific sample size assigned for mosquito vectors collection since currently there is no formal guidance on thresholds for the filarial DNA prevalence that is consistent with LF elimination targets. Therefore, the desire was to collect as many mosquitoes as possible in order to analyze a relatively high number of mosquito pools as done in a previous study to assess the LF status in Sri Lanka [26].

### Parasitological surveys

#### Community sensitization, participant recruitment and sampling

For the community-based survey, public meetings (known locally as *baraza*) were held with adult residents in each study village to explain the aims of the study including the procedures, risks and benefits of participation. The meetings were conducted using Kiswahili language and participants given an opportunity to ask questions and provide suggestions. Mobilization of the community members to participate in the *baraza* was done by the village elders on behalf of the local assistant chief. Complete household lists were obtained from the village elders and approximately 55 households randomly selected using the RAND function in Excel. The locations of the selected households were drawn on sketch maps prepared with the help of the village elders and trained local field assistants. The trained local field assistants recruited two adults (a male and a female) from each selected household. Households with adult members who refused to participate were replaced by a nearby household.

For the school-based surveys, meetings were held with the parents and/or legal guardians of children enrolled in classes 1–3 (targeting children aged 6–8 years) to explain the aims of the study, including the procedures, risks and benefits of participation. Adult participants were provided with stool collection containers on the day prior to sample collection so that they could collect fresh stool samples in the morning in the privacy of their homes. They were asked to come with the stool samples at an agreed collection site where fresh urine samples were also collected. The registered schoolchildren were provided with both stool and urine collection containers in their respective schools on the day of sample collection. Prior arrangements were made with the school administration so that the stool and urine samples could be collected when the children were released from the classrooms for a midmorning break.

#### Laboratory examination for STH infections and intestinal SCH

Stool samples were microscopically tested for the presence of the eggs of different species of intestinal helminths using the quantitative Kato-Katz method as previously described [27,28]. Briefly, two 41.7 mg stool smears were made from each stool sample on the day of sample collection for examination and counting of intestinal helminths eggs. The smears were first examined for hookworm eggs within 1 hour and next day for other intestinal helminths eggs. Approximately 10% of the positive and negative Kato-Katz thick smear slides were randomly selected and re-examined for quality control purposes by an experienced laboratory scientist, on a day-to-day basis. The same experienced laboratory scientist performed quality control of all the Kato-Katz and urine filtration microscopy.

#### Laboratory examination for urogenital SCH

Urine samples were collected in wide-mouth plastic containers between 9:00 AM and 12:00 noon and 10 ml aliquots used for examination of schistosome eggs using the urine filtration technique as previously described [23,27]. Briefly, the 10 ml urine specimens were aliquoted using disposable syringes and filtered through 12 μm polycarbonate membrane filters at the sample collection site. The filters were carefully placed on labeled microscope glass slides and stored in slide boxes for later reading under a light microscope.

#### Laboratory examination for LF

One hundred microlitres (100 μl) of finger prick whole blood was collected to test for CFA using the immunochromatographic (ICT) test (Binax Inc., Maine, USA). The results were read exactly at 10 minutes, following the instructions of the manufacturer. All individuals who tested positive for CFA using the ICT test were further tested for MF in nighttime finger prick blood samples (100 μl) collected between 20:00 and 24:00 hours. The night-time finger prick blood samples were collected using heparinized capillary tubes and transferred into tubes containing 0.9 ml of 3% acetic acid and mixed gently as previously described [20]. The samples were transported to the nearby field laboratory where they were stored at ambient temperature until the following day. The counting chamber method [29] was used for microscopic examination of MF in the nighttime blood specimens. The ICT test readings and counting chamber slides were confirmed by a second reader and an experienced laboratory scientist, respectively for quality control purposes.

#### LF mosquito survey

Host seeking mosquitoes were collected using CDC-LTs and Ifakara-TTs in 10 houses per village towards the end of a rainy season (months) in order to ensure that large numbers were caught for MX. The houses for mosquito collection were purposively selected based on some convenience criteria, including walls made of mud, roofs thatched with either grass or coconut leaves (*makuti*), and with open eaves. Collection of mosquitoes was done for 10 consecutive nights in each of the months of December 2012 and January 2013 (i.e., total of 20 nights in each house). The light trap was placed near the bed area within the house, approximately 1.5 metres from the floor to catch the host-seeking mosquitoes attempting to feed on occupants from 6:00 PM to 6:00 AM. The technicians and field assistants setting up the traps confirmed that an occupant(s) would sleep in the bed area at night.

The Ifakara-TT was erected in an optimal location outside the house selected for the CDC-LT setting. The design of the Ifakara-TT is described in details by Govella et al [19]. In brief, the Ifakara-TT is rectangular canvas tent containing six funnel-like entrances for mosquitoes and inner small apertures tilted to an angle so that mosquitoes have to fly upward to enter the trap. Such baffled entrance structures are known to increase the probability that mosquitoes do not exit once inside traps [30]. The base of the trap is made of thick polyvinylchloride sheeting, which protects against rough substrates and surface water. Trained field assistants slept overnight inside the Ifakara-TT and any trapped host-seeking mosquitoes were collected in the morning and transported to the field laboratory in the nearby Langobaya health facility. Trained laboratory technicians sorted the mosquitoes while still fresh and females were identified to genus level using morphological characteristics [31,32]. The mosquitoes were sorted by community and trap type and stored in pools of up to 20 in tubes containing silica gel desiccant. The preserved mosquitoes were transported to the Eastern and Southern Africa Centre of International Parasite Control (ESACIPAC) laboratory in KEMRI at the end of the field work for real-time PCR analysis.

#### Molecular analysis

The mosquitoes were re-sorted in pools of up to 5 mosquitoes to increase the sample size, which yielded to a total of 1,055 pools. Total genomic DNA was extracted using the Qiagen DNeasy kit (Cat No. 69506, Qiagen, Valencia, CA) as per the manufacturer’s instructions. The samples were eluted in final volume of 100 μl of the elution (AE) buffer. The eluted DNA samples were stored at -20°C until real-time PCR was performed and thereafter transferred to -80°C for long term storage.

A real-time PCR protocol designed to amplify a 1674 bp “long DNA repeat (LDR)” sequence of *W*. *bancrofti* (LDR; GenBank accession no. AY297458) in the pools was performed as described previously [33]. Briefly, the assay was performed using 12.5 μl of TaqMan master mix (Applied Biosystems) along with 450 nmol/L of each primer and 125 nmol/L probe in a final volume of 25 μl. Positive and negative control samples were included in each PCR assay. All the PCR reactions were carried out in duplicate in microtiter plates with an Applied Biosystems 7500 Standard Real-Time PCR system.

#### Data analysis

Data analysis was conducted using IBM SPSS^®^ Statistics software (Version 21, Armonk, NY: IBM Corp.) for descriptive statistics. The Chi-Square (χ²) test was used to compare categorical variables, for example, LF, STH and SCH infection status. The SPSS software was used to compute geometric means after log-transformation of the data. Data were tested for normal distribution using the Shapiro-Wilk test. Since the data were not normally distributed and occurred in a few individuals, infection intensities for STH (eggs per gram, EPG) and *S*. *haematobium* (eggs per 10 ml of urine) were compared using the non-parametric independent samples for the positive individuals only. The Kruskal-Wallis and Mann-Whitney U tests were used to compare the intensities among the villages and between males and females, respectively. The intensity categories for STH and *S*. *haematobium* infections were computed as per the thresholds proposed by a WHO Expert Committee in 1987 [34,35].

The PoolScreen software (Version 2.0.2) was used to determine the maximum likelihood estimate and 95% confidence intervals (Clopper-Pearson Fiducial method) of *W*. *bancrofti* infection prevalence in mosquito pools [36]. A P value lower than 0.05 was considered statistically significant.

### Ethical statement

This study received approval following review by the Scientific Steering and Ethical Review Committees of the Kenya Medical Research Institute (KEMRI SSC protocol No.1970) and the Liverpool School of Tropical Medicine Research Ethics Committee (LSTM protocol No. 10.84 RS). The Sub-county education and health officers, local leaders, teachers and parents were sensitized about the study in the area. The study was conducted following the tenets of the Helsinki Declaration. An information sheet was provided and read to all eligible participants during study enrollment. A written informed consent was obtained from adults and parents, and legal guardians or caretakers of children less than18 years old. Written assent was also obtained from children aged 12 years and above. No study participants’ identifiers were included in the dataset used for the analyses in order to ensure privacy.

### Inclusivity in global research

Additional information regarding the ethical, cultural, and scientific considerations specific to inclusivity in global research is included in the (S1 Checklist).

## Results

### Parasitological surveys

A total of 436 adults from the four study villages were enrolled in the community-based survey (Table 1). There were significantly more female (n = 303, 69.5%) compared with male (133, 30.5%) participants (P < 0.05). The median age of male participants was higher at 44 years (range, 13–80) compared to 37 years (range, 13–92) for females (P < 0.05). Out of the 436 community members enrolled into the study, 389 and 431 individuals provided stool and urine samples for STH and *S*. *haematobium* microscopy, respectively. A total of 471 schoolchildren aged between 4 to 11 years (median age of 9 years) were enrolled into a school-based survey, but 11 children did not provide a blood sample. Of the 460 children who provided blood samples, 227 (49.7%) were females and 233 (50.7%) were males. No significant difference was found in age distribution between the schools (P = 0.051).

**Table 1:** Demographic characteristics of individuals recruited for parasitological surveys of helminthic infections in four villages along River Galana/ Sabaki in Kilifi County, coastal Kenya.

	Jilore	Marikano	Magongoloni^§^	Mkondoni	Total
**Community arm**					
No. examined (n)	92	109	119	116	436
Sex					
No. female (%)*	70 (76.1)	75 (68.8)	80 (67.2)	78 (67.2)	303 (69.5)
No. male (%)	22 (23.9)	34 (31.2)	39 (32.8)	38 (32.8)	133 (30.5)
Age (yrs)					
Median	44	43	42	30	40
Range (min-max)	16–92	15–76	15–82	15–75	15–92
**School arm**					
No. examined (n)	120	119	116	116	471
Sex					
No. female (%)	62 (51.7)	60 (50.4)	56 (48.3)	57 (49.1)	235 (49.9)
No. male (%)	58 (48.3)	59 (49.6)	60 (51.7)	59 (50.9)	236 (50.1)
Age (yrs)					
Median	9	9	9	9	9
Range (min-max)	5–10	5–11	4–10	7–10	4–11

*Significantly more females than males participated in the community based human survey (P < 0.05).

^**§**^The name of the school is Maji Langobaya, which includes children from outside the study village.

Table 2 provides a summary of the results of the prevalence of the 3 infections among school children and community members in the study setting. The random re-examination of a subset of the Kato-Katz thick smears and the ICT antigen test results did not identify any substantial discrepancies in the reported readings. *T*. *trichiura* was the most common STH among the schoolchildren ranging from 2.6% to 8.8%. Conversely, hookworms were most common among the adult population ranging from 7.4% to 14.3%. The intensities of the infections are shown in Table 3. The STH infections among the schoolchildren were mostly low-intensity except one *A*. *lumbricoides* infection case with moderate-intensity in Mkondoni school. Among the adult population, 4 cases with *A*. *lumbricoides* (19%) out of the 21 positives were moderate-intensity infections while the rest were light-intensity. All *T*. *trichiura* infections were light-intensity. Out of the 45 cases with hookworms, 42 (93.3%), 2 (4.4%) and 1 (2.2%), were light-, moderate-, and heavy-intensity infections, respectively. The differences in prevalence and intensity of STH infections across the communities and between males and females were not statistically significant.

**Table 2:** Prevalence of helminthic NTDs among schoolchildren and community members in 4 villages along River Galana/ Sabaki in Kilifi County, coastal Kenya.

	Jilore	Marikano	Magongoloni	Mkondoni	Total	
	No. pos/No. exam (%)	No. pos/No. exam (%)	No. pos/No. exam (%)	No. pos/No. exam (%)	No. pos/No. exam (%)	*P value
**School arm**						
*S*. *haematobium*	9/117 (7.7)	2/118 (1.7)	4/116 (3.4)	2/115 (1.7)	17/466 (3.6)	0.047
*A*. *lumbricoides*	0/117 (0)	4/114 (3.5)	5/114 (4.4)	6/114 (5.3)	15/459 (3.3)	0.119
*T*. *trichiura*	5/117 (4.3)	10/114 (8.8)	3/114 (2.6)	9/114 (7.9)	27/459 (5.9)	0.153
Hookworm	3/117 (2.6)	5/114 (4.4)	3/114 (2.6)	5/114 (4.4)	16/459 (3.5)	0.779
LF	1/118 (0.8)	1/117 (0.9)	0/113 (0)	0/112 (0)	2/460 (0.4)	0.588
**Community arm**						
*S*. *haematobium*	0/90 (0)	1/108 (0.9)	4/119 (3.4)	7/114 (6.1)	12/431 (2.8)	0.031
*A*. *lumbricoides*	2/84 (2.4)	8/99 (8.1)	6/111 (5.4)	5/95 (5.3)	21/389 (5.4)	0.408
*T*. *trichiura*	5/84 (6.0)	4/99 (4.0)	2/111 (1.8)	6/95 (6.3)	17/389 (4.4)	0.370
Hookworm	12/84 (14.3)	13/99 (13.1)	13/111 (11.7)	7/95 (7.4)	45/389 (11.6)	0.478
LF	7/92 (7.6)	2/109 (1.8)	8/117 (6.8)	6/115 (5.2)	23/433 (5.3)	0.248

*Pearson Chi-Square test (χ²).

**Table 3:** Intensities of helminthic NTDs among schoolchildren and community members in 4 villages along River Galana/ Sabaki in Kilifi County, coastal Kenya.

	Jilore	Marikano	Magongoloni	Mkondoni	Total	
	GM (95% CI)	GM (95% CI)	GM (95% CI)	GM (95% CI)	GM (95% CI)	*P value
**School arm**						
*S*. *haematobium*	30.7 (7.4–125.4)	43.3 (36.0–52.0)	138.1 (51.0–406.0)	24.7 (16.0–38.0)	184.3 (51.9–244.0)	1.000
*A*. *lumbricoides*	0	92.3 (12.0–840.0)	79.0 (12.0–352.9)	197.6 (37.8–1413.6)	118.8 (46.1–335.5)	0.669
*T*. *trichiura*	50.2 (12.0–248.5)	51.0 (25.2–95.7)	68.0 (24.0–156.0)	43.3 (21.7–79.8)	49.7 (32.3–75.5)	0.982
Hookworm	94.7 (36.0–492.0)	64.8 (17.0–287.5)	443.9 (228.0–864.0)	99.1 (60.0–218.4)	114.0 (60.7–218.4)	0.193
**Community arm**						
*S*. *haematobium*	0	30.0 (30.0–30.0)	31.1 (3.0–186.0)	11.8 (3.9–32.0)	17.6 (7.4–37.6)	0.264
*A*. *lumbricoides*	1219.8 (252.0–5904.0)	133.2 (31.2–783.9.0)	197.2 (78.7–567.2)	1700.0 (388.8–7819.0)	337.4 (143.6–771.2)	0.073
*T*. *trichiura*	24.6 (12.0–47.5)	42.8 (24.0–108.0)	93.0 (48.0–180.0)	25.1 (14.3–46.5)	33.0 (22.9–49.5)	0.178
Hookworm	339.0 (154.6–730.0)	140.0 (43.6–432.5)	61.9 (31.4–129.1)	134.4 (51.2–323.0)	139.1 (85.4–222.7)	0.063

*Independent samples Kruskal-Wallis Test.

GM: Geometric means (eggs per gram of faeces, EPG for STH and eggs per 10 ml of urine for intestinal SCH) were calculated for positive individuals only.

The prevalence of *S*. *haematobium* infection was statistically different across the schools (P = 0.047) and villages (P = 0.031). However, the intensities of the infection were not statistically different in either of the two arms nor between males and females. Out of 12 adults with urogenital schistosomiasis, 11 (91.7%) were light-intensity infections. Intestinal schistosomiasis was not detected in the area. Overall, urogenital schistosomiasis was more prevalent among the schoolchildren compared to the adult population. Interestingly, the village (Jilore) that had the highest prevalence of the disease among schoolchildren (7.7%) had the lowest prevalence among the adult population (0%). Out of the 17 children found to have *S*. *haematobium* eggs in their urine, 9 and 8 cases were light- and heavy-intensity, respectively.

The overall prevalence of LF infection among the adult population in the area was 5.3% and ranged from 1.8% to 7.6% across the 4 villages, but the difference was not statistically significant (Table 2). Males had higher prevalence estimate of LF (9.2%) compared to females (3.7%) (P < 0.05). All the 23 CFA-positive individuals were MF-negative in confirmatory night blood testing. The median age of the CFA positives was 49 years (range, 17−70 years). Two children, both male and aged 10 years, were found to be positive for filarial antigen but none was found to have MF in nighttime blood samples.

### Mosquito sampling

**Fig 1:**
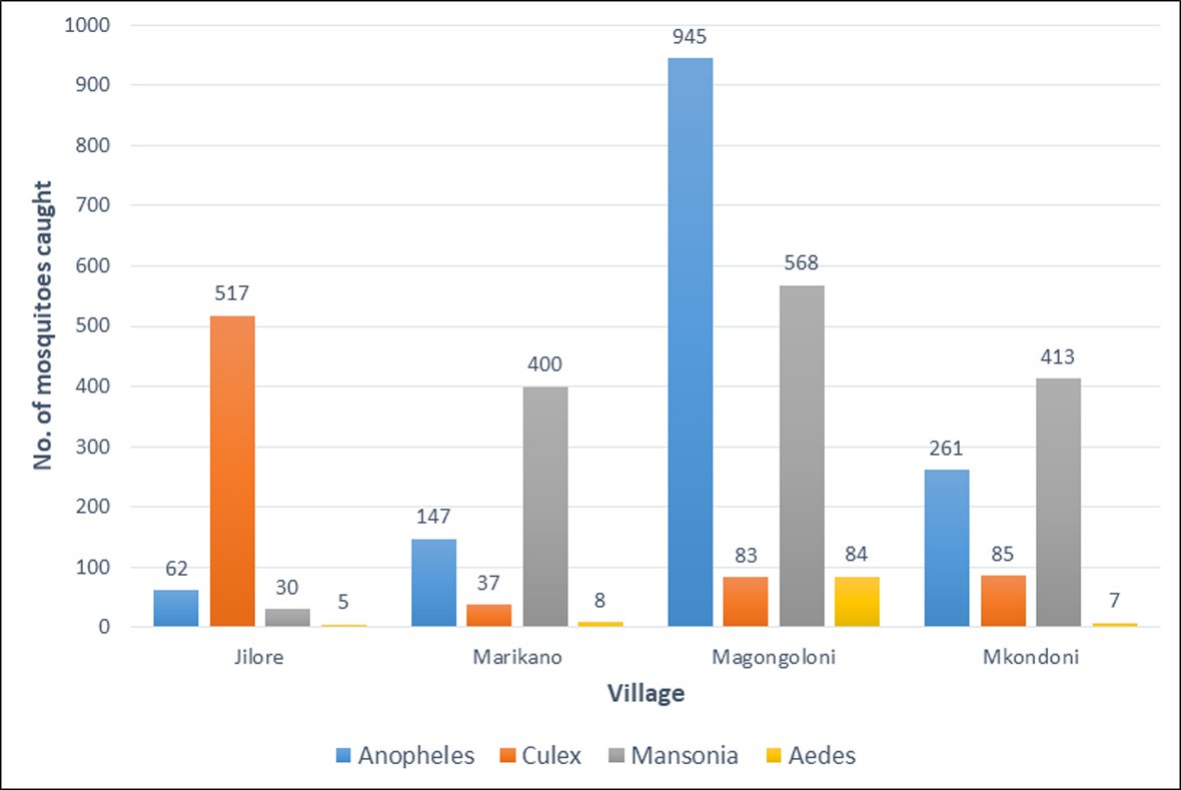
Mosquito species trapped using two methods in the four villages along River Galana/ Sabaki in Kilifi County, coastal Kenya.

A total of 3,652 mosquitoes were caught in the four villages, belonging to 4 genera (Fig 1). A significantly greater number (1,680) of mosquitoes were captured in Magongoloni village (P < 0.05) compared to other villages, and the main genus was *Anopheles*. *Culex* mosquitoes were abundant in Jilore compared to the other villages. Overall, the CDC-LT collected almost twice (n = 2,423) as many mosquitoes compared to the Ifakara-A TT (n = 1,229), P < 0.05. The mosquitoes caught were stored in pools of 1 to 5 mosquitoes yielding a total of 1,055 pools (mean pool size of 3.46, 95% CI: 3.36–3.56). There were 694 pools from the CDC-LT and 361 pools from the Ifakara-TT (Table 4). The CDC-LT was significantly more efficient in trapping mosquitoes belonging to *Anopheles* and *Mansonia* genera. However, the Ifakara-TT was more efficient in collection of *Culex* mosquitoes.

**Table 4:** Total number of mosquito pools belonging to four genera analyzed by real-time PCR.

Trap type/ genus	Village				Total
	Jilore	Marikano	Magongoloni	Mkondoni	
**CDC light trap**					
*Anopheles* (%)	21 (6.1)	39 (11.3)	216 (62.4)	70 (20.2)	346 (100)
*Culex* (%)	32 (35.2)	14 (15.4)	28 (30.8)	17 (18.7)	91 (100)
*Mansonia* (%)	11 (4.9)	62 (27.4)	102 (45.1)	51 (22.6)	226 (100)
*Aedes* (%)	1 (3.2)	3 (9.7)	23 (74.2)	4 (12.9)	31 (100)
**Sub-total (%)**	**65 (9.4)**	**118 (17.0)**	**369 (53.2)**	**142 (20.5)**	**694 (100)**
**Ifakara A tent trap**					
*Anopheles* (%)	10 (15.9)	12 (19.0)	18 (28.6)	23 (36.5)	63 (100)
*Culex* (%)	105 (79.5)	4 (3.0)	6 (4.5)	17 (12.9)	132 (100)
*Mansonia* (%)	5 (3.0)	52 (31.7)	45 (27.4)	62 (37.8)	164 (100)
*Aedes* (%)	0 (0.0)	0 (0.0)	2 (100.0)	0 (0.0)	2 (100)
**Sub-total (%)**	**120 (33.2)**	**68 (18.8)**	**71 (19.7)**	**102 (28.3)**	**361 (100)**
**Grand total (%)**	**185 (17.5)**	**186 (17.6)**	**440 (41.7)**	**244 (23.1)**	**1,055 (100)**

### Detection of *Wuchereria bancrofti* DNA by real-time PCR

Out of 1,055 pools tested by real-time PCR, one was positive for the presence of *W*. *bancrofti* DNA. The overall maximum likelihood estimate was 0.00027 and 95% CI for the infection prevalence was 0.0000069–0.0015224. The one positive pool consisted of mosquitoes belonging to the *Anopheles gambiae* complex collected from Magongoloni village where 440 pools of this mosquito species were tested. The maximum likelihood estimate for the infection was 0.00059 in Magongoloni with a 95% CI 0.000017–0.003315.

## Discussion

Preventive anthelminthic chemotherapy is an effective public health tool that uses available anthelminthic drugs either alone or in combination to prevent morbidity caused by these NTDs, or in some cases, to eliminate the diseases [11,37]. Since several helminthic NTDs are co-endemic within affected communities in sub-Saharan Africa, provision of integrated interventions should be considered whenever feasible. Another important consideration for these co-endemic helminthic NTDs is whether integrated epidemiological surveys are feasible. This study provides evidence on the possibility of integrating STH and SCH into school- and community-based LF surveys which is important for increasing the efficiency of monitoring and/or surveillance. The WHO NTD road map 2021–2030 calls for a shift from disease-specific to integrated approaches through cross-cutting activities that intersect multiple diseases [1]. The road map further advocates that monitoring, evaluation and reporting should be integrated for all relevant endemic NTDs. Integrated surveillance of helminthic NTDs during the post-intervention phase could be an efficient and cost-effective approach particularly if resources become limited.

The finding that the overall prevalence of STH infections was generally low, and did not vary markedly between villages might be due to the impact of the mass treatment provided to control and eliminate LF in the area. A cross-sectional study of a sentinel village on Alor Island in Eastern Indonesia also found that MDA consisting of DEC and albendazole provided against LF (*Brugia timori)* resulted in reduction in STH infections [38]. It is not surprising that the annual albendazole provided with DEC had only modest effects on *T*. *trichiura* prevalence, because this species is known to be less susceptible to albendazole than the other STH species [39,40]. The presence of relatively high prevalence of hookworm and *A*. *lumbricoides* infections among the adult population corroborates the results from a previous study in Kwale County in the southern coastal region in Kenya [28]. The finding calls for the consideration of implementation of intervention programmes that include the adult population who if left out might remain a reservoir for the infection.

The observation that the prevalence of urogenital schistosomiasis was statistically different among the schools and villages underscores the focal distribution of the disease. Similar observations from other settings have led to a call for the consideration of innovative mapping approaches of schistosomiasis endemicity and implementation of interventions at administration level(s) lower than the district (sub-county in Kenya). For example, the mapping of all schools in an implementation unit, termed precision mapping, has been one such suggested approach [41]. The finding that children in one village had urogenital schistosomiasis while the disease was absent among the adult population is interesting. The observation corroborates the observation that in communities with access to several water sources, even individuals within the same risk group may be endowed with differential infection risk because of their specific water-contact patterns [42]. As such, a comprehensive description of heterogeneity in schistosomiasis transmission also requires an account of individual preferences related to water contacts [43].

The detection of up to 7.6% prevalence of CFA and a mosquito pool containing filarial DNA may be an indication of possible ongoing transmission of LF infection. Thus, the results of this study may provide a justification for the additional rounds of MDA given in the area. However, the failure to detect microfilariae in the night blood specimens of the antigen positive individuals suggests that the transmission, if any, was at very low level. Nonetheless, annual mass treatment with DEC and albendazole was restarted in 2015 and to date six consecutive MDA rounds have been administered. In June-July 2021, the national programme for elimination of LF conducted pre- transmission assessment survey (pre-TAS) in Kilifi County and the preliminary results obtained indicate that antigenemia is currently below the 2% critical threshold in all implementation units in the county and thus they may be considered eligible for LF transmission assessment survey (TAS) as recommended by WHO [12]. It is expected that the consecutive annual MDA since 2015 may have reduced the community microfilaria density below the threshold that would yield infective larvae in *Anopheles* and *Culex* mosquitoes which are major vectors of LF in the setting [44]. In June 2022, the national NTD programme conducted an LF TAS survey which integrated STH, SCH and tungiasis (jiggers). Comparison of the results of the surveillance with those of this study will provide insights on the current status of the infections in the setting.

Mosquito surveys were effectively carried out without the use of the ethically questionable human-landing catch, the gold standard for sampling human biting mosquitoes [45]. Overall, the CDC-LT showed the best performance in catching *Anopheles* and *Mansonia* mosquitoes whereas the Ifakara-TT was more efficient in trapping *Culex* species. Thus, the selection of trapping method may be guided by the dominant species and a combination of traps may be considered in areas where different vector species occur. The differences in mosquito abundance among the villages is likely due to spatial heterogeneity in vector breeding habitats between the four villages. For example, *Culex* mosquitoes were captured in greater number in one village (Jilore) compared to the others. The *Culex* mosquito is associated with stagnant waters rich in organic material mainly found in urban and/or peri-urban areas and may be an indication of anthropogenic changes in the environmental status in this village. It is noteworthy that substantial numbers of *Mansonia* mosquitoes were caught during this study although none was found to have filarial DNA. Studies in Ghana have found *Mansonia* species to be vectors of *W*. *bancrofti* [46].

The finding that only one of the 1,055 pools of mosquitoes was positive, suggests that mosquitoes may pick up a few microfilariae from a low-density human host, but probably incapable of yielding infective larvae. The WHO has recognized the use of MX and antifilarial antibody testing as potential future surveillance strategies [12]. However, practical guidelines for systematic sampling of vector mosquitoes for MX are currently lacking [47]. Thus, this study provided key information on potential LF vectors and additional evidence for the consideration of MX as a supplemental monitoring and surveillance approach for LF transmission. It also provided information on alternative mosquito trapping tools, which are also still lacking 10 years on from this study and highlights the need for further investment in the development of practical and sustainable trapping tools to improve MX activities.

There are several limitations to this study. One was that the baseline prevalence and intensity of STH and SCH infections were not measured prior to the start of the MDA programme. Thus, it is not possible to provide the exact impact of albendazole provided as part of the LF intervention on the STH infections. Although the study demonstrated that integrated surveillance of STH, SCH and LF is possible and provided key data to inform local intervention activities, it remains to be shown how practical this could be under programmatic conditions. This study is one of the few studies published or presented at NTD meetings in the last decade highlighting the value of an integrated approach to better understand the epidemiological situation in Kenya, and has importance and relevancy elsewhere. A further limitation is related to the fact that interventions to eliminate LF as a public health problem are often given at implementation units which in many countries are administrative levels known as districts or their equivalent. Thus, this study would have given more generalizable results if it was conducted at the implementation unit level (sub-county in Kenya). The inability to provide estimation of cost-effectiveness of the integrated surveillance reported in this study is also considered a study limitation.

Limitations related to the entomological work included that other mosquito collection methods were not employed at the time, which may have an impact on the number and diversity of the mosquitoes collected. For example, the CDC gravid trap has been shown to be an efficient method for the trapping of *Culex* mosquitoes [48]. In the sub-Saharan Africa region *Culex* and *Anopheles* mosquitoes are reported as the principal vectors of LF, although there is minimal evidence that the former species contribute to the transmission of the disease in West Africa [49]. A previous study in the area reported that mosquitoes belonging to the genus *Anopheles* are principal vectors in rural areas in this setting [24]. Consequently, this study did not anticipate collection of substantial numbers of *Culex* mosquitoes since the villages are primarily rural. In contrast, a good number of *Culex* mosquitoes were caught in all the villages in this study. Thus, gravid traps should be considered as a trap for LF vector mosquitoes when conducting future studies in coastal Kenya. The lack of information on blood-feeding status of the vector mosquitoes trapped (unfed, fed and gravid) is another study limitation. The information may be useful in providing additional knowledge on the connection between mosquito blood-feeding behaviour and the presence of filarial larvae which may have a direct impact on the transmission of LF infection. Also, this study was conducted during an unusually wet rainy season, which may have affected the mosquito composition and abundance. It would be desirable to assess the effectiveness of the traps over multiple years and across different seasons of the weather.

## Conclusions

This study demonstrated that integrated epidemiological survey using standard parasitological and entomological methods is an efficient approach that can provide useful information on co-endemic parasitic diseases which could help direct interventions and surveillance activities. Further studies, however, may be necessary to assess the role of MX as a monitoring and evaluation, as well as a surveillance strategy for LF elimination programmes post-MDA.

## Supporting information

S1 Checklist Checklist with additional information regarding the ethical, cultural, and scientific considerations specific to inclusivity in global research. (DOCX)
